# In silico analysis of serum miRNA profiles in seronegative and seropositive rheumatoid arthritis patients by small RNA sequencing

**DOI:** 10.7717/peerj.15690

**Published:** 2023-07-27

**Authors:** Xiao-Hong He, Yun-Ting Xiao, Wen-Ying Chen, Mao-Jie Wang, Xiao-Dong Wu, Li-Yan Mei, Kai-Xin Gao, Qing-Chun Huang, Run-Yue Huang, Xiu-Min Chen

**Affiliations:** 1 State Key Laboratory of Dampness Syndrome of Chinese Medicine, The Second Affiliated Hospital of Guangzhou University of Chinese Medicine, Guangzhou, China; 2 Second Clinical Medical College, Guangzhou University of Chinese Medicine, Guangzhou, China; 3 Department of Rheumatology, The Second Affiliated Hospital of Guangzhou University of Chinese Medicine, Guangzhou, China; 4 Guangdong-Hong Kong-Macau Joint Lab on Chinese Medicine and Immune Disease Research, Guangzhou, China; 5 Guangdong Provincial Key Laboratory of Chinese Medicine for Prevention and Treatment of Refractory Chronic Diseases, Guangzhou, China

**Keywords:** Seronegative rheumatoid arthritis, Seropositive rheumatoid arthritis, Small RNA sequencing, miRNA, Biomarker

## Abstract

Rheumatoid arthritis (RA) is a refractory autoimmune disease, affecting about 1% of the world’s population. RA is divided into seronegative RA and seropositive RA. However, biomarkers for discriminating between seronegative and seropositive RA have not been reported. In this study, we profiled serum miRNAs in seronegative RA patients (N-RA), seropositive RA patients (P-RA) and healthy controls (HC) by small RNA sequencing. Results indicated that compared with HC group, there were one up-regulated and four downregulated miRNAs in N-RA group (fold change ≥ 2 and *P* value < 0.05); compared with P-RA group, there were two up-regulated and four downregulated miRNAs in N-RA group; compared with HC group, there were three up-regulated and four downregulated miRNAs in P-RA group. Among them, the level of hsa-miR-362-5p in N-RA group was up-regulated compared with that in HC group and P-RA group, and the level of hsa-miR-6855-5p and hsa-miR-187-3p in P-RA group was upregulated compared with that in N-RA group and HC group. Validation by qPCR confirmed that serum hsa-miR-362-5p level was elevated in N-RA group. Subsequently, by analyzing the target genes using RNAhybrid, PITA, Miranda and TargetScan and functions of differential miRNAs utilizing Gene Ontology (GO) and Kyoto Encyclopedia of Genes and Genomes (KEGG), we found that the target genes and molecular pathways regulated by miRNAs in seronegative RA and seropositive RA were roughly the same, and miRNAs in these two diseases may participate in the occurrence and development of diseases by regulating the immune system. In conclusion, this study revealed the profiles of serum miRNAs in seronegative and seropositive RA patients for the first time, providing potential biomarkers and targets for the diagnosis and treatment of seronegative and seropositive RA.

## Introduction

Rheumatoid arthritis (RA) is a systemic inflammatory disease characterized by multiple joint involvement and extraarticular manifestations, including accelerated cardiovascular disease (Scott, Wolfe & Huizinga, 2010). The disease affects about 1% of the world population, and the incidence rate of women is 2 to 3 times that of men (Smolen et al., 2018). RA is incurable and can lead to persistent pain, deformity and disability, resulting in significant personal and social costs (Smolen, Aletaha & McInnes, 2016). As an autoimmune disease, RA induces production of some specific factors, including rheumatoid factor (RF) and anti-cyclic citrullinated peptide antibody (ACPA). Combined detection of RF and ACPA can significantly improve the diagnostic specificity and sensitivity of RA (Gavrila, Ciofu & Stoica, 2016). However, some patients without the presence of RF and ACPA are diagnosed as seronegative RA, whereas those with RF and ACPA are diagnosed as seropositive RA (Pratt & Isaacs, 2014). These two types of RA cause autoimmune dysfunction through different mechanisms, thus the follow-up treatment methods are also different (Ajeganova & Huizinga, 2015). At present, in addition to rheumatoid factor and anti-cyclic citrullinated peptide antibody, there are no other effective markers to discriminate between seronegative and seropositive RA. However, the sensitivity and specificity of protein detection limit the diagnostic accuracy of seronegative and seropositive RA. Therefore, the current study detected the profile of miRNAs in the serum of patients with either seronegative or seropositive RA, thereby sorting out differential miRNAs between seronegative and seropositive RA.

MicroRNA (miRNA) is a kind of non-coding RNA with a length of only 22–25 nucleotides, which are expressed in multiple organs and circulatory system (including blood) (Backes, Meese & Keller, 2016). Serum miRNAs have been identified as reliable biomarkers and therapeutic targets for RA. For instance, serum miR-126-3p, let-7d-5p, miR-431-3p, miR-221-3p, miR-24-3p and miR-130a-3p are elevated in RA patients and considered as biomarkers for early progression of RA (Cunningham et al., 2021). Besides, the level of serum exosomal miR-1915-3p is increased in clinical-remission RA patients, which might be a promising biomarker for RA activity (Lim et al., 2020). Moreover, serum miR-10 level is raised in patients with high RA activity and it negatively associates with serum interleukin 35 concentration, suggesting that serum miR-10 might be biomarkers and therapeutic targets for RA (Paradowska-Gorycka et al., 2022). However, the effects of serum miRNAs on discriminating between seronegative and seropositive RA remains unknown.

To date, small RNA sequencing is wildly used in screening biomarkers for RA and autoimmune diseases in serum miRNAs. For example, Dunaeva et al. (2018), identified that serum miR-223-3p and miR-16-5p are potential biomarkers for early RA by small RNA sequencing. Moreover, Ebrahimkhani et al. (2017) found a unique miRNA signature in serum exosomes to differentiate relapsing-remitting from progressive disease in multiple sclerosis. In addition, Hamada et al. (2015), revealed that serum miR-150-5p is a candidate biomarkers for diagnosis of autoimmune pancreatitis. Therefore, the small RNA sequencing was used to detect the serum miRNA profiles in patients with seronegative or seropositive RA and identify differential miRNAs, which might be candidate biomarkers for discriminating between seronegative and seropositive RA. Next, results of small RNA sequencing were validated by qPCR. Then the enrichment analysis of GO terms and KEGG pathways for target genes of differential miRNAs were conducted to explore potential functions of differential miRNAs.

## Materials and Methods

### Ethical statement

All the experimental procedures were approved by the Institutional Review Board of Guangdong Provincial Hospital of Chinese Medicine (#BF2018-088-01) and conducted in accordance with the Declaration of Helsinki. Written informed consent had been obtained from all participants in this study.

### Patients

In this study, nine healthy people (HC group), 12 patients with seronegative RA (N-RA group) and nine patients with seropositive RA (P-RA group) were recruited. All patients were recruited from the rheumatology clinic of the Second Affiliated Hospital of Guangzhou University of Chinese Medicine (Guangdong Provincial Hospital of Chinese Medicine, Guangzhou, China). RA patients were enrolled according to the following inclusion criteria: (1) RA was diagnosed according to the 2009 American College of Rheumatology (ACR) diagnostic criteria; (2) patients were classified as grade I, II or III according to the classification of American Rheumatic Association in 1987; (3) patients agreed to participate in the study and signed the informed consent. Then patients were diagnosed as seronegative or seropositive RA based on the presence of RF and ACPA.

### Serum collection

Fasting venous blood were drawn and centrifugated to separate serum. Subsequently, the separated serum was stored at −80 °C for detection.

### Construction, sequencing and miRNA identification of small RNA library

Total RNA was extracted from serum by TRIzol (Thermo-Fisher-Scientific, Waltham, MA, USA). Next, RNAs with the length of 18–30 nucleotides were enriched utilizing polyacrylamide gel electrophoresis. Subsequently, the 5′ and 3′ end adaptors were ligated to the RNA. Then the ligation products were reverse transcribed, and 140–160 base products were collected to construct small RNA library and sequenced by Illumina HiSeqTM 4000 in Beijing Genomics Institute (Shenzhen, Guangdong, China).

In order to get high-quality clean reads, raw reads were analyzed through in-house Perl scripts. After eliminating the low-quality reads with >10% poly-N sequences and <5% Phred score, all the clean reads were aligned with small RNAs using GeneBank database and Rfam database (11.0) to remove rRNA, scRNA, snoRNA, snRNA and tRNA. At the same time, all clean reads were also aligned with human reference genome (Grch37) by TopHat (v2.0.9) software.

Subsequently, all clean reads were blasted in the miRBase database (version 21) to identify the known miRNAs in the clean reads. Besides, all unannotated tags were aligned with the human reference genome (Grch37) to explore new candidate miRNAs according to the predicted genome location and hairpin structure by Mireap_v0.2 software.

### MiRNA profiles

The levels of miRNAs were standardized to transcripts per million (TPM) using the following formula:

$\rm TPM= {actual\ miRNA\, quantity} / {total\, clean\, data\, using\, the\, following\, formula} \times 10^6$

In addition, the total miRNA heat map was created to show the miRNA levels in different groups and to cluster miRNAs with similar patterns.

### Analysis of differential miRNA

Based on the miRNA levels, the differential miRNAs among different groups were determined by the following formula:

${p(x|y)}={\left(\frac{N_2}{N_1}\right)^y}{\frac{(x+y)!}{{x!y!{}\left(1+\frac{N_2}{N_1}\right)}^{x+y+1}}}\matrix{{C\left(y\leq y_{\rm min}|x\right)=\sum\limits_{y=0}^{y\leq y_{min}}p(y|x)}\\ {D\left(y\geq y_{\rm max}|x\right)=\sum\limits_{y\geq y_{\rm max}}^{\infty}p(y|x)}}$

MiRNAs with a fold change ≥2 and *P* value < 0.05 were considered as significantly differential miRNAs.

### Validation of small RNA sequencing data by qPCR

QPCR assays were performed to confirm the reliability of the small RNA sequencing data according to previous studies (Xu et al., 2018; Zhong et al., 2014). First, reverse transcription for miRNAs were performed by the miRcute miRNA First-Strand cDNA Synthesis Kit (Tiangen, Beijing, China). Next, miRNA levels were normalized to the level of 5sRNA using the ΔΔCT method. The sequences of primers used in this study were listed as follows: 5sRNA forward: 5′-AAGCCTACAGCACCCGGTAT-3′, reverse: 5′-GTAACGCCCGATCTCGTCT-3′.

Fold change (log2) values of miRNAs were presented as mean $\pm$ standard error of the mean (SEM). Moreover, statistical analyses were performed on fold change values using ordinary one-way ANOVA, marked as *P < 0.05 compared with other groups.

### Prediction of miRNA target genes

Based on the sequences of miRNAs, candidate target genes of miRNAs were predicted by RNAhybrid (v2.1.2), PITA, Miranda (v3.3a) and TargetScan. Furthermore, the intersection of target genes predicted by the above software and the website were more reliable. In addition, the miRNA-target gene network was constructed by using Cytoscape software (v3.6.0).

### Enrichment analysis of GO and KEGG pathways for miRNA target genes

Based on the Gene Ontology (http://www.geneontology.org/) database, all the target genes of differential miRNAs were matched to GO terms, and the number of genes matching each GO term was also counted. In addition, GO terms with significant enrichment compared to human genome background were defined *via* hypergeometric test analysis. Besides, KEGG was used for molecular pathway enrichment analysis to identify pathways in which target genes of differential miRNAs are involved.

## Results

### Identification of miRNAs in serum

Thirty small RNA libraries including 12 N-RA samples, nine HC samples and nine RA samples were constructed and sequenced to demonstrate the miRNA profiles. Clean reads were obtained after filtering low quality reads. Next, rRNA, scRNA, snoRNA, snRNA and tRNA were removed from the clean reads through comparing with GenBank and Rfam (11.0). Then the clean reads were mapped to the human reference genome (Grch37) by using TopHat. Results indicated that 180, 201 and 216 known miRNAs (Table S1) and 15, 11 and 18 new miRNAs (Table S2) were found in N-RA, HC and P-RA samples, respectively.

### Prolife of differential miRNAs in serum

**Figure 1:**
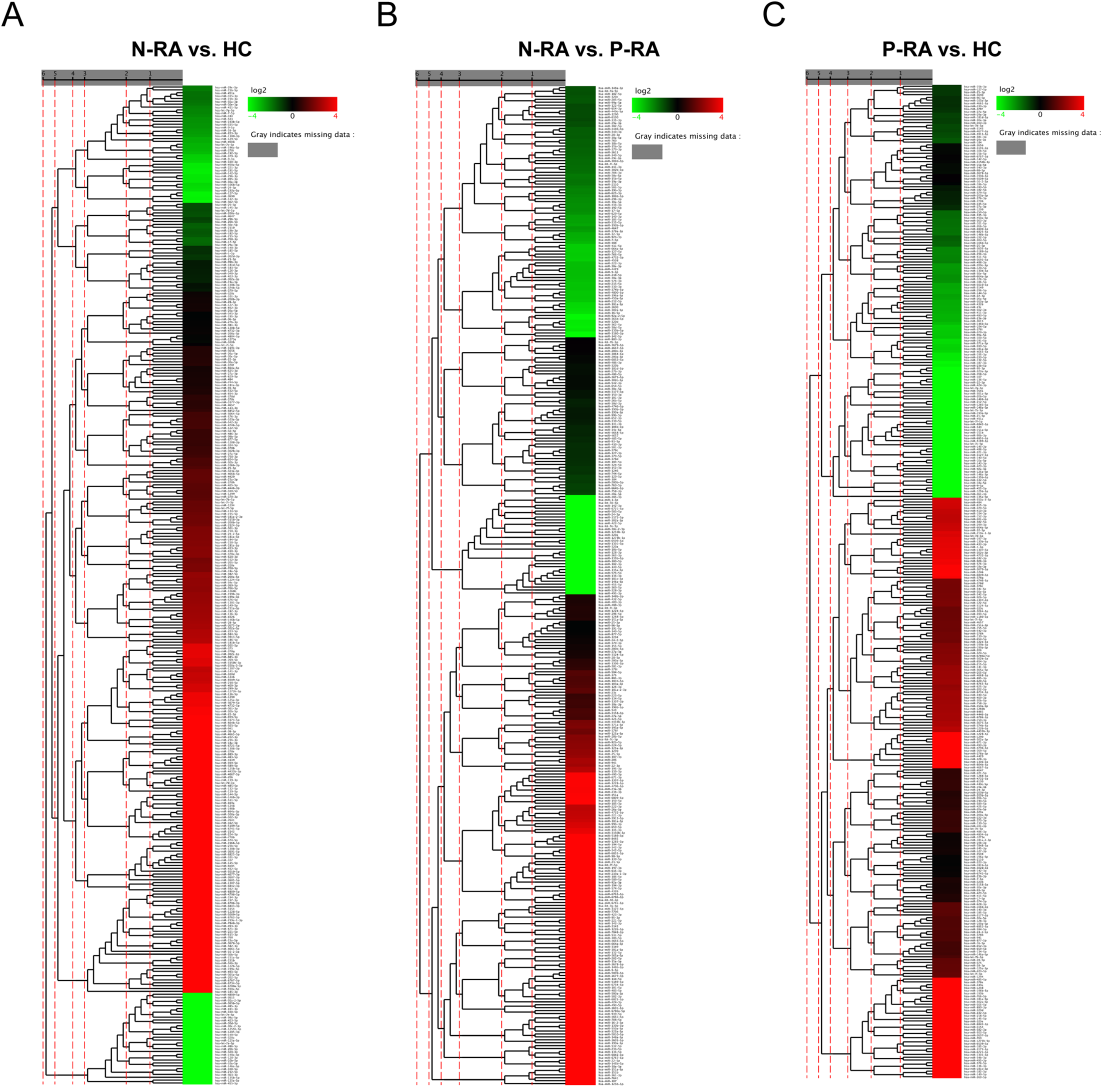
Characteristics of miRNA levels between different groups. The heat map showing miRNA levels between N-RA group and HC group (A), N-RA group and P-RA group (B) and P-RA group and HC group (C). HC, healthy control; N-RA, seronegative RA; P-RA, seropositive RA.

Levels of serum miRNAs were determined by reading count and TPM analysis. Colors from blue to red stand for z-score got through the dimensionality reduction of FPKM value and reveal decreasing miRNA levels in each group. Compared with HC group, five differential miRNAs (one up-regulated and four down-regulated) were found in N-RA group, including hsa-miR-362-5p, hsa-miR-4429, hsa-miR-378e, hsa-miR-302c-3p and hsa-miR-378g (Fig. 1A and Table 1). Compared with P-RA group, there were six differential miRNAs (two up-regulated and four down-regulated) in N-RA group, including hsa-miR-362-5p, hsa-miR-708-3p, hsa-miR-6741-5p, hsa-miR-3127-5p, hsa-miR-6855-5p and hsa-miR-187-3p (Fig. 1B and Table 2). Compared with HC group, seven differential miRNAs (three up-regulated and four down-regulated) were identified in P-RA group, including hsa-miR-6855-5p, hsa-miR-187-3p, hsa-miR-371a-5p, hsa-miR-302a-3p, hsa-miR-320e, hsa-miR-218-5p and hsa-miR-378e (Fig. 1C and Table 3). Among them, the level of hsa-miR-362-5p in N-RA group was upregulated compared with that in HC group and P-RA group, the level of hsa-miR-378e in N-RA group and P-RA group was downregulated compared with that in HC group, and the level of hsa-miR-6855-5p and hsa-miR-187-3p in P-RA group was upregulated compared with those in N-RA group and HC group (*P* < 0.05). These results suggest that serum hsa-miR-362-5p might be used as a diagnostic marker for seronegative RA, while serum hsa-miR-6855-5p and hsa-miR-187-3p might be promising diagnostic markers for seropositive RA.

**Table 1:** Differently expressed miRNA (N-RA *vs* HC).

miRNA	Group		Up-/Down-regulated	Log2 (Fold change)
	N-RA	HC		
hsa-miR-362-5p	0.151394564	0.01	Up	3.920241501
hsa-miR-4429	0.097494943	0.283607444	Down	−1.540496108
hsa-miR-378e	0.01	0.539587039	Down	−5.753783791
hsa-miR-302c-3p	0.01	0.122616762	Down	−3.616084305
hsa-miR-378g	0.01	0.122423043	Down	−3.613803223

**Table 2:** Differently expressed miRNAs (N-RA *vs* P-RA).

miRNA	Group		Up-/Down-regulated	Log2 (Fold change)
	N-RA	P-RA		
hsa-miR-362-5p	0.129890228	0.01	Up	3.69922099
hsa-miR-708-3p	0.03360368	0.01	Up	1.74861923
hsa-miR-6741-5p	0.01	0.810320123	Down	−6.340420063
hsa-miR-3127-5p	0.01	0.946841448	Down	−6.565050956
hsa-miR-6855-5p	0.01	0.184964376	Down	−4.20917553
hsa-miR-187-3p	0.01	0.052250413	Down	−2.385442428

**Table 3:** Differently expressed miRNAs (P-RA *vs* HC).

miRNA	Group		Up-/Down-regulated	Log2 (Fold change)
	P-RA	HC		
hsa-miR-6855-5p	0.239985	0.01	Up	4.584875185
hsa-miR-187-3p	0.148407	0.01	Up	3.891482393
hsa-miR-371a-5p	0.108831	0.01	Up	3.444023416
hsa-miR-302a-3p	0.017142	0.360278	Down	−4.393518258
hsa-miR-320e	0.005714	0.919273	Down	−7.329863854
hsa-miR-218-5p	0.01	0.911274	Down	−6.509812533
hsa-miR-378e	0.01	0.720331	Down	−6.170588065

### Validation of small RNA sequencing data

**Figure 2:**
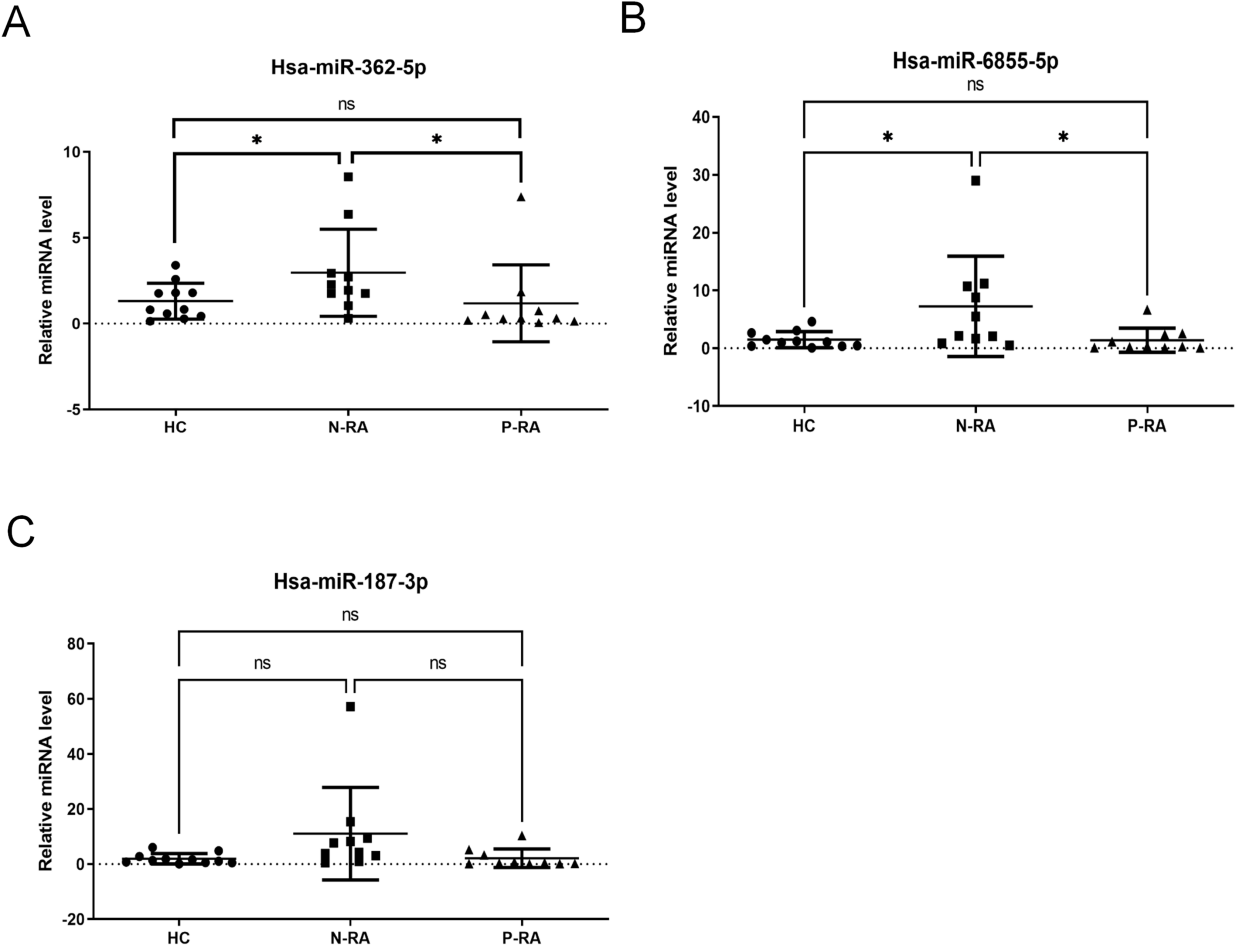
Validation of small RNA sequencing data. (A–C) Levels of serum hsa-miR-362-5p, hsa-miR-6855-5p and hsa-miR-187-3p determined by RT-PCR. HC, healthy control; N-RA, seronegative RA; P-RA, seropositive RA. *P < 0.05.

Subsequently, levels of serum hsa-miR-362-5p, hsa-miR-6855-5p and hsa-miR-187-3p were validated in 11 HC samples, 10 N-RA samples and 10 P-RA samples by qPCR. There was a 165.3% increase in serum hsa-miR-362-5p level in N-RA group compared to that in HC group, while there was a 178.4% increase in serum hsa-miR-362-5p level in N-RA compared to that in P-RA group (Fig. 2A). Besides, there was a 575.6% increase in serum hsa-miR-6855-5p level in N-RA compared to that in HC group, while there was a 585.1% increase in serum hsa-miR-6855-5p level in N-RA compared to that in P-RA group (Fig. 2B). Moreover, there was no difference of serum hsa-miR-187-3p level among three groups (Fig. 2C). Above results showed that the level of serum hsa-miR-362-5p was consistent with that determined by small RNA sequencing (Fig. 2A). However, levels of serum hsa-miR-6855-5p and hsa-miR-187-3p were opposite to results of small RNA sequencing (Figs. 2B and 2C). Thus, these data suggested that serum hsa-miR-362-5p should be the promising diagnostic marker for seronegative RA.

### Prediction of differential miRNA target genes

**Figure 3:**
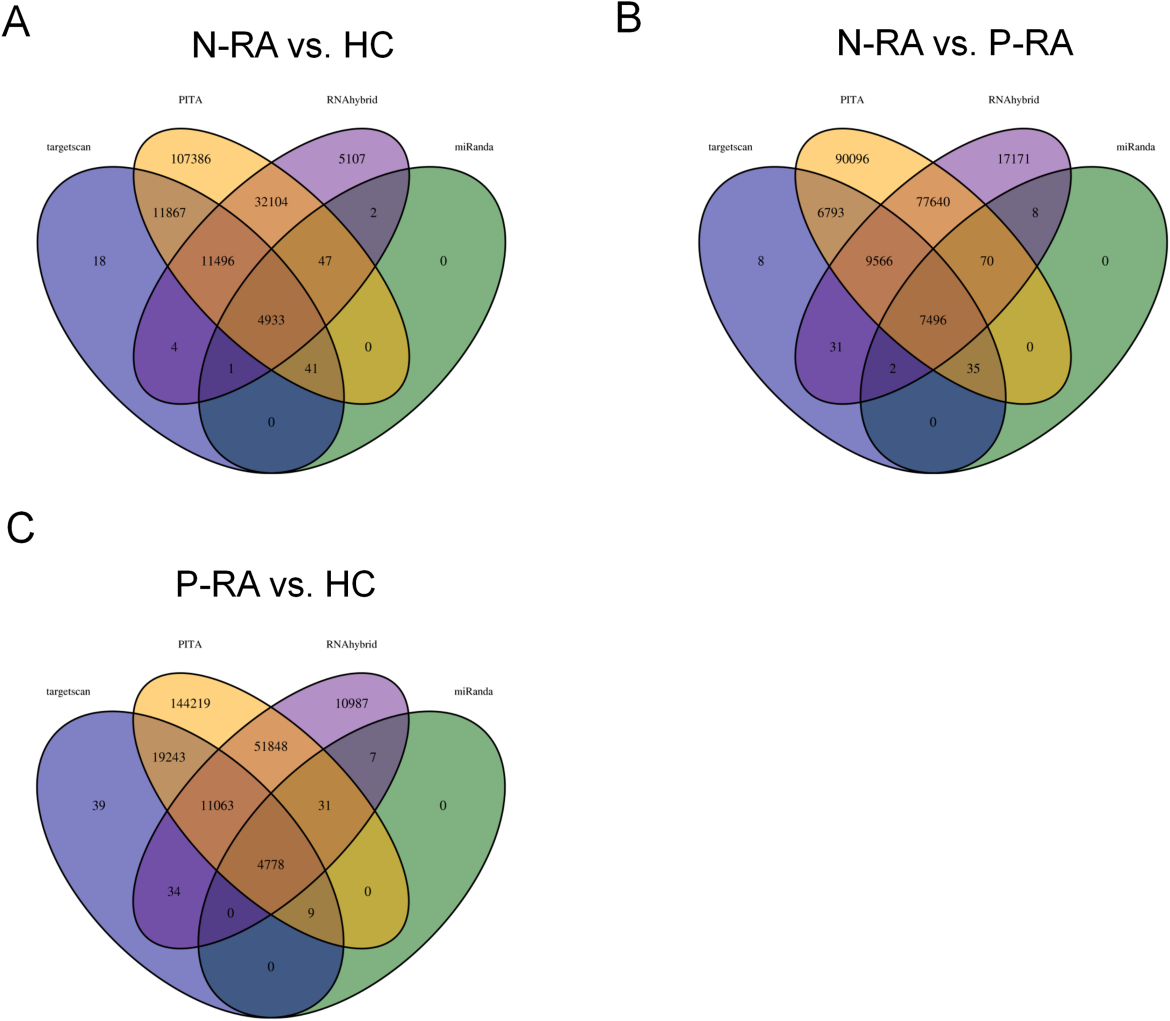
Numbers of differently expressed miRNA target genes. The target genes of differently expressed miRNAs were analyzed by four databases, and the number of common target genes was counted using Wayne diagram. (A) The number of differently expressed miRNA target genes between N-RA group and HC group calculated by Wayne diagram. (B) The number of differently expressed miRNA target genes between P-RA group and N-RA group calculated by Wayne diagram. (C) The number of differently expressed miRNA target genes between P-RA group and HC group calculated by Wayne diagram. HC, healthy control; N-RA, seronegative RA; P-RA, seropositive RA.

**Figure 4:**
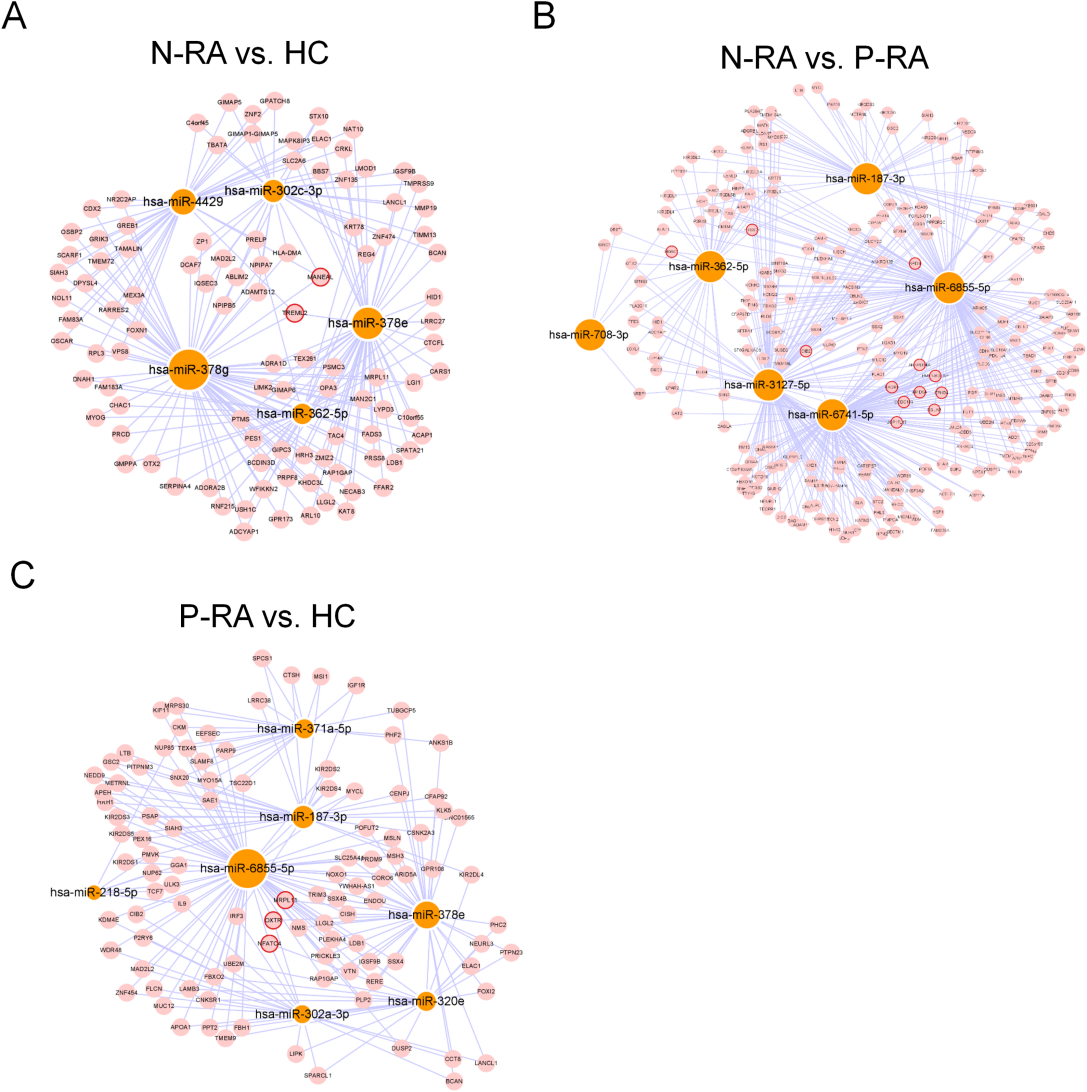
MiRNA-mRNA regulatory network between differently expressed miRNAs and target genes. The regulatory relationship between differently expressed miRNAs and target genes was elucidated through miRNA-mRNA regulatory network. (A) The miRNA-mRNA regulatory network of differently expressed miRNAs and target genes between N-RA group and HC group. (B) The miRNA-mRNA regulatory network of differently expressed miRNAs and target genes between N-RA group and P-RA group. (C) The miRNA-mRNA regulatory network of differently expressed miRNAs and target genes between P-RA group and HC group. HC, healthy control; N-RA, seronegative RA; P-RA, seropositive RA.

MiRNA often plays a vital role in biological process by regulating the expression of target genes. To explore the effects of miRNAs on seronegative and seropositive RA, target genes of differential miRNAs were predicted to understand potential functions of differential miRNAs. Results showed that there were 4,933 recognized target genes of differential miRNAs between N-RA and HC group (Fig. 3A), 7,496 recognized target genes of differential miRNAs between N-RA and P-RA group (Fig. 3B), and 4,778 recognized target genes of differential miRNAs between P-RA and HC group (Fig. 3C). Among them, target genes of differential miRNAs between N-RA and HC groups included dysferlin (DYSF), myosin light chain 2 (MYL2), glucosylceramidase beta (GBA), tumor necrosis factor (TNF), CD4 molecule (CD4), mannosidase endo-alpha like (MANEAL), triggering receptor expressed on myeloid cells like 2 (TREML2) (Fig.4A and Table S3). Besides, target genes of differential miRNAs between N-RA and P-RA group included phosphatase, orphan 2 (PHOSPHO2), GBA, TNF, CD79b molecule (CD79B), CD4, calcium and integrin binding family member 2 (CIB2), progestin and adipoQ receptor family member 6 (PAQR6) (Fig.4B and Table S3). Moreover, target genes of differential miRNAs between P-RA and HC group included steroidogenic acute regulatory protein (STAR), MYL2, GBA, TNF, CD4, mitochondrial ribosomal protein L11 (MRPL11), oxytocin receptor (OXTR) (Fig.4C and Table S3). Results showed that most target genes of differential miRNAs among three groups were consistent. In addition, miRNA-mRNA networks revealed that target genes could be regulated by both upregulated miRNAs and downregulated miRNAs (Figs. 4A–4C and Table S3).

### Functional analysis of differential miRNAs

**Figure 5:**
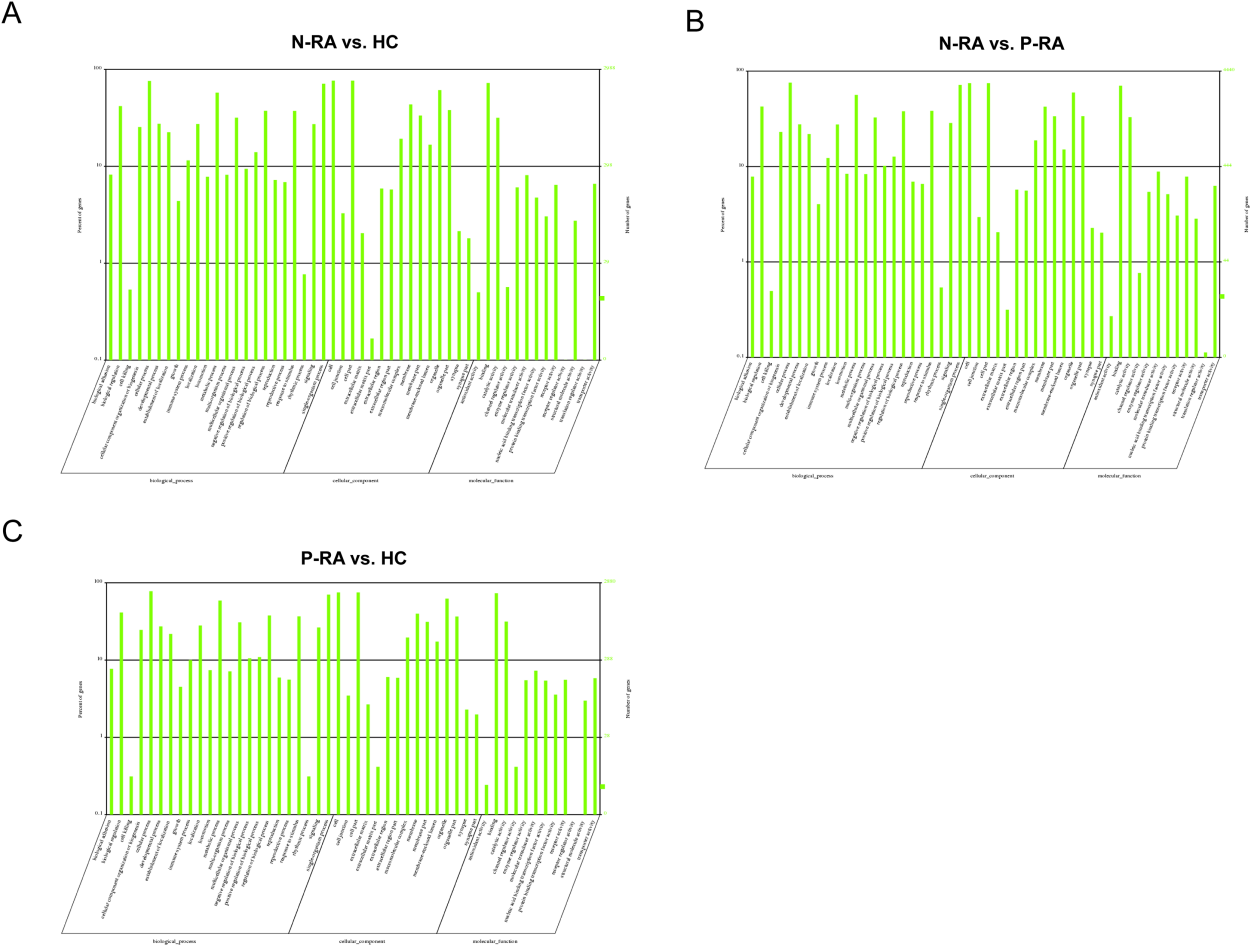
GO enrichment analysis for target genes of miRNAs with differently levels. (A) The GO enrichment histograms and GO terms for target genes of differently expressed miRNAs between N-RA group and HC group. (B) The GO enrichment histograms and GO terms for target genes of differently expressed miRNAs between N-RA group and P-RA group. (C) The GO enrichment histograms and GO terms for target genes of differently expressed miRNAs between P-RA group and HC group. HC, healthy control; N-RA, seronegative RA; P-RA, seropositive RA.

To further clarify the function of differential miRNAs, GO and KEGG pathway enrichment were utilized to analyze the physiological processes and molecular pathways contributed to the regulation of target genes. GO enrichment analysis showed that target genes of differential miRNAs between N-RA and HC group were mainly involved in binding, catalytic activity, molecular transduction activity, transport activity, receptor activity (Fig. 5A). Besides, target genes of differential miRNAs between N-RA and P-RA group mainly contributed to catalytic activity, molecular transduction activity, receptor activity, transport activity and nucleic acid transcription factor activity (Fig. 5B). Furthermore, target genes of differential miRNAs between P-RA and HC group were mainly involved in binding, catalytic activity, molecular transduction activity, transport activity and nucleic acid transcription factor activity (Fig. 5C). These results indicated that molecular functions of differential miRNAs among three groups were almost identical.

**Figure 6:**
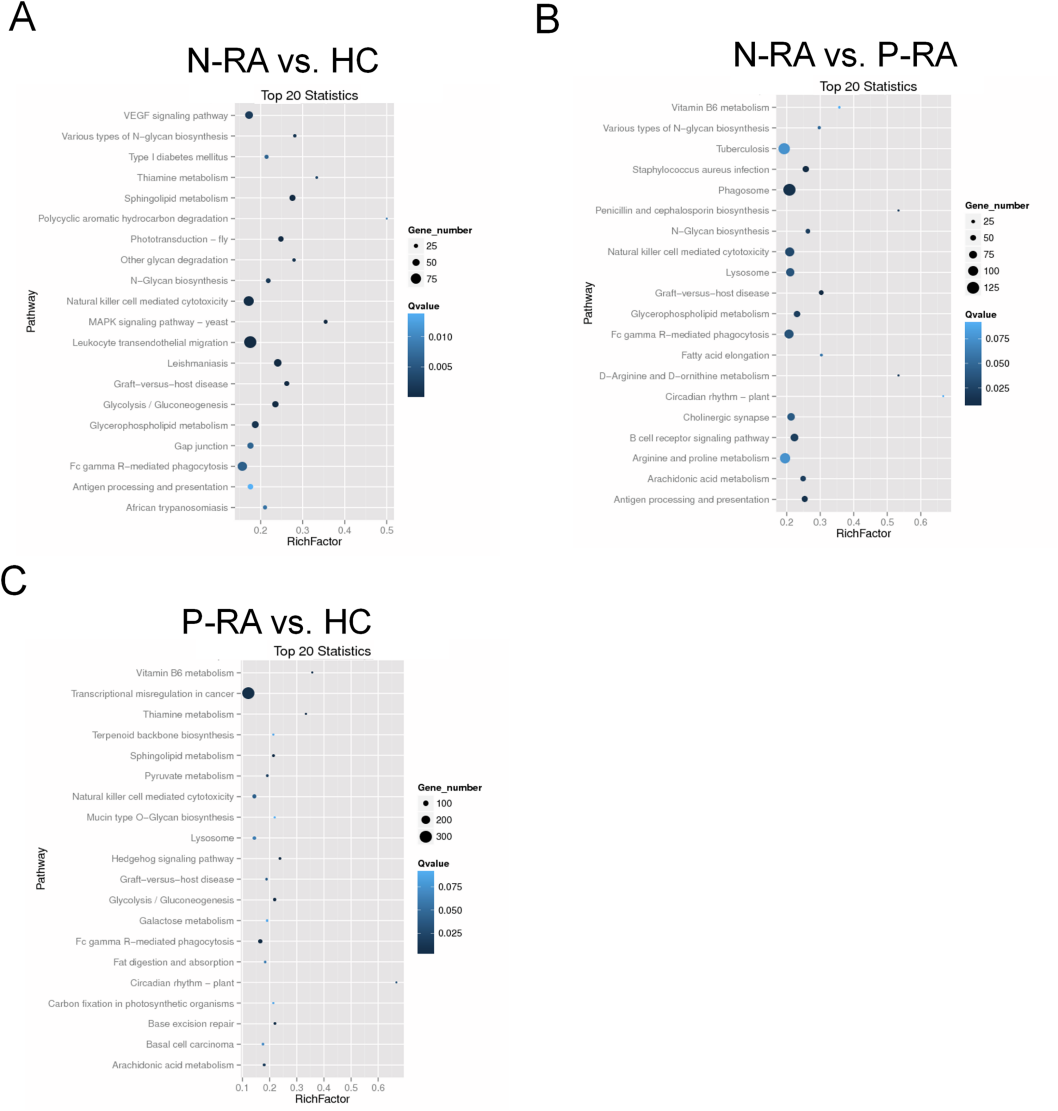
KEGG pathway enrichment analysis for target genes of miRNAs with differently levels. The KEGG pathway enrichment scatter plots for target genes of differently expressed miRNAs between N-RA group and HC group (A), N-RA group and P-RA group (B), P-RA group and HC group (C). HC, healthy control; N-RA, seronegative RA; P-RA, seropositive RA.

In addition, KEGG enrichment analysis showed that target genes of differential miRNAs between N-RA and HC group were mainly involved in VEGF signaling pathway, natural killer cell mediated cytotoxicity, leukocyte endothelial migration, N-glycan biosynthesis, sphingolipid metabolism, and antigen processing presentation (Fig. 6A). Besides, target genes of differential miRNAs between N-RA and P-RA group were mainly involved in vitamin B6 metabolism, natural killer cell mediated cytotoxicity, N-glycan biosynthesis, B-cell receptor signaling pathway, and antigen processing and presentation (Fig. 6B), while target genes of differential miRNAs between P-RA and HC group mainly contributed to transcriptional misregulation in cancer, vitamin B6 metabolism, natural killer cell mediated cytotoxicity, sphingolipid metabolism, and N-glycan biosynthesis (Fig. 6C). These results revealed that differential miRNAs among the three groups regulated similar molecular pathways.

## Discussion

In this study, we identified the serum miRNA profiles of seronegative RA patients (N-RA), healthy people (HC), and seropositive RA patients (P-RA). A total of 180, 201, and 216 known miRNAs and 15, 11, and 18 novel miRNAs were found in N-RA samples, HC samples, and P-RA samples, respectively. In addition, compared with HC group, there were five differential miRNAs in N-RA group, including hsa-miR-362-5p, hsa-miR-4429, hsa-miR-378e, hsa-miR-302c-3p and hsa-miR-378g. Compared with P-RA group, there were six differential miRNAs in N-RA group, including hsa-miR-362-5p, hsa-miR-708-3p, hsa-miR-6741-5p, hsa-miR-3127-5p, hsa-miR-6855-5p and hsa-miR-187-3p. Compared with HC group, there were seven differential miRNAs in P-RA group, including hsa-miR-6855-5p, hsa-miR-187-3p, hsa-miR-371a-5p, hsa-miR-302a-3p, hsa-miR-320e, hsa-miR-218-5p and hsa-miR-378e. Among them, the level of hsa-miR-362-5p in N-RA group was up-regulated compared with that of HC group and P-RA group, the level of hsa-miR-378e in N-RA group and P-RA group was down-regulated compared with those in HC group, and the levels of hsa-miR-6855-5p and hsa-miR-187-3p in P-RA group were up-regulated compared with those of N-RA group and HC group (*P* < 0.05). Validation by qPCR revealed that serum hsa-miR-362-5p should be the promising diagnostic marker for seronegative RA.

Among the differential miRNAs, only hsa-miR-218-5p was reported to be associated with RA. Recent studies have shown that hsa-miR-218-5p regulates the proliferation, apoptosis, autophagy and oxidative stress of rheumatoid arthritis synovial fibroblasts by targeting Kruppel like factor 9 (KLF9) and activating JAK2/STAT3 signaling pathway (Chen et al., 2021). In addition, hsa-miR-218-5p induces osteogenic differentiation of rheumatoid arthritis synovial fibroblasts by regulating ROBO1/DKK-1 pathway. However, the role of other differential miRNAs in RA (including seronegative and seropositive) is unknown. Therefore, this study is the first time to identify differential miRNAs in the serum of patients with seronegative RA (N-RA), healthy people (HC) and patients with seropositive RA (P-RA). It is also the first time to propose that hsa-miR-362-5p could be used as a diagnostic marker for seronegative RA, while hsa-miR-6855-5p and hsa-miR-187-3p could be used as diagnostic markers of seropositive RA.

Although the relationship between most differential miRNAs and RA is still unclear, target genes of differently expressed miRNAs are related to RA or autoimmune diseases. For example, TNF is a recognized diagnostic indicator of RA. Now it also has become a clinical treatment target of RA, and anti-TNF therapy has been an effective method for the treatment of RA (Aletaha & Smolen, 2018; Radner & Aletaha, 2015). Besides, CD4 positive T helper cells (Th cells) play a crucial role in the development of RA by regulating adaptive immune response (Yang et al., 2019), while CD4 positive T cells participate in the regulation of immune metabolism in early and late RA (Weyand & Goronzy, 2017). Moreover, anti-CD79b antibody could induce the cell-free immune response of B cells to resist autoimmune diseases (Hardy et al., 2014). *Dysferlin* deletion is also closely related to the occurrence and development of autoimmune diseases (Matsubara et al., 2001; Selva-O’Callaghan et al., 2006). Therefore, these miRNAs may participate in the occurrence and development of seronegative and seropositive RA by regulating the immune system. However, this study did not analyze specific target genes related to seronegative or seropositive RA, which will be solved in the follow-up study.

Above studies have shown that the target genes regulated by differential miRNAs are associated with RA or autoimmune diseases, so we continued to explore the molecular pathways which miRNAs may participate in. Similar to the target genes, most of the molecular pathways regulated by the differential miRNAs are related to autoimmune diseases and immunity. For example, CD84-mediated signaling induces autoimmune diseases by regulating natural killer cell-mediated cytotoxicity (Cuenca et al., 2019). Antigen processing and presentation plays an important role in the occurrence and development of most autoimmune diseases (Unanue, Turk & Neefjes, 2016). Moreover, MAP kinase kinase kinase kinases 1 (MAP4K1) regulates the phosphorylation or ubiquitination of downstream target proteins through B cell receptor signal to regulate the immune response and influence the occurrence and development of autoimmune diseases (Chuang, Wang & Tan, 2016). Therefore, we further believe that differential miRNAs participate in the occurrence and development of seronegative and seropositive RA by regulating the immune system.

Meanwhile, this study also found that most target genes of differential miRNAs, molecular functions of differential miRNA target genes and molecular pathways involved in differential miRNA target genes among the three groups are identical. These results indicated that differential miRNAs in both seronegative and seropositive RA regulate similar target genes and molecular pathways. However, the profile of miRNAs may be different in seronegative or seropositive RA, resulting in differences in biological functions. For instance, a target gene is downregulated in seronegative RA by upregulated miRNAs, whereas it is upregulated in seropositive RA by downregulated miRNAs. Therefore, we will explore these target genes in the follow-up study, which might be used as specific therapeutic targets for seronegative and seropositive RA.

However, there were some limitations in the present study. For instance, the novel biomarker (serum hsa-miR-362-5p) in larger sample sizes should be confirmed using AUC value. Besides, serum levels of targets of hsa-miR-362-5p also should be detected. Moreover, the effects of hsa-miR-362-5p and its targets on seronegative RA should be identified by *in vitro* and *in vivo* experiments. Therefore, we would further confirm that serum hsa-miR-362-5p should be a novel biomarker for seronegative RA, detect serum levels of targets of hsa-miR-362-5p and explore the effects of hsa-miR-362-5p and its targets on seronegative RA in our future study.

## Conclusion

This study revealed the serum miRNA profiles in patients with seronegative RA, healthy people and patients with seropositive RA. Results indicated that serum hsa-miR-362-5p should be the promising diagnostic marker for seronegative RA. These findings would provide diagnosis biomarker and specific targets for treatment of seronegative and seropositive RA.

## Supplemental Information

10.7717/peerj.15690/supp-1 Supplemental Information 1 Supplementary Table S1.

10.7717/peerj.15690/supp-2 Supplemental Information 2 Supplementary Table S2.

10.7717/peerj.15690/supp-3 Supplemental Information 3 Supplementary Table S3.

10.7717/peerj.15690/supp-4 Supplemental Information 4 Raw data.

10.7717/peerj.15690/supp-5 Supplemental Information 5 Statistical analyse for hsa-miR-187-3p.

10.7717/peerj.15690/supp-6 Supplemental Information 6 Statistical analyse for hsa-miR-362-5p.

10.7717/peerj.15690/supp-7 Supplemental Information 7 Statistical analyse for hsa-miR-6855-5p.

## Additional Information and Declarations

### DNA Deposition

The following information was supplied regarding the deposition of DNA sequences: The sequences are available at SRA: PRJNA929076.

### Data Availability

The following information was supplied regarding data availability: The sequencing data are available at SRA: PRJNA929076. The raw measurements are available in the Supplemental Files.
