# Supplementary figures and images for: In silico analysis of serum miRNA profiles in seronegative and seropositive rheumatoid arthritis patients by small RNA sequencing
Source: PeerJ. 2023 Jul 27;11:e15690. doi: 10.7717/peerj.15690 (PMC10387234; doi:10.7717/peerj.15690)

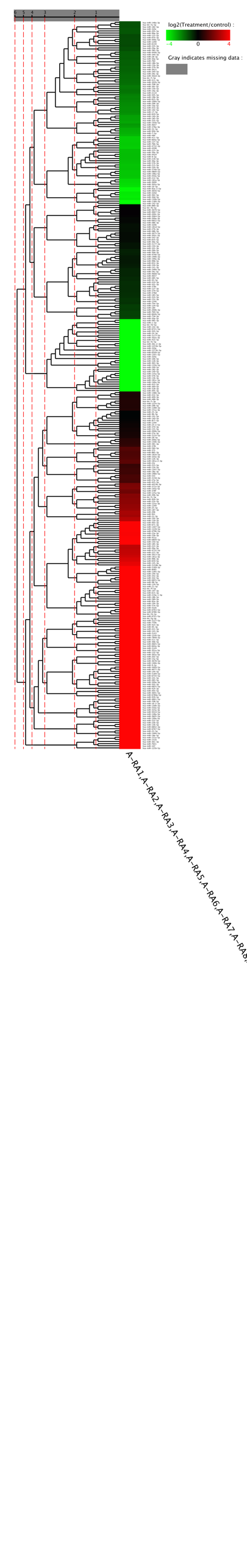

Supplement: Supplemental Information 4 [file peerj-11-15690-s004.zip › Raw data/Figure 1/Figure 1 A.png]

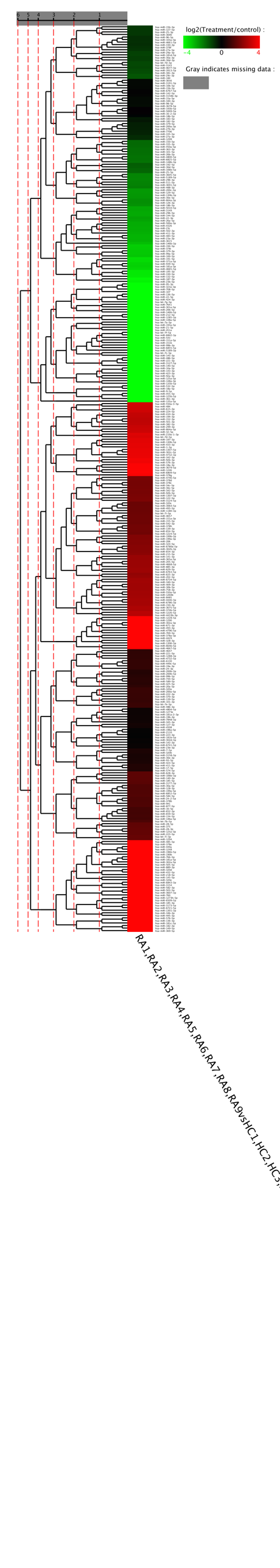

Supplement: Supplemental Information 4 [file peerj-11-15690-s004.zip › Raw data/Figure 1/Figure 1 B.png]

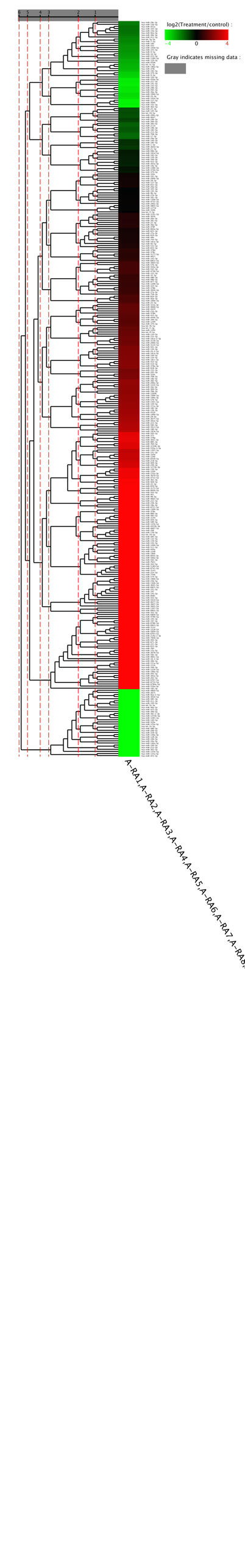

Supplement: Supplemental Information 4 [file peerj-11-15690-s004.zip › Raw data/Figure 1/Figure 1 C.png]

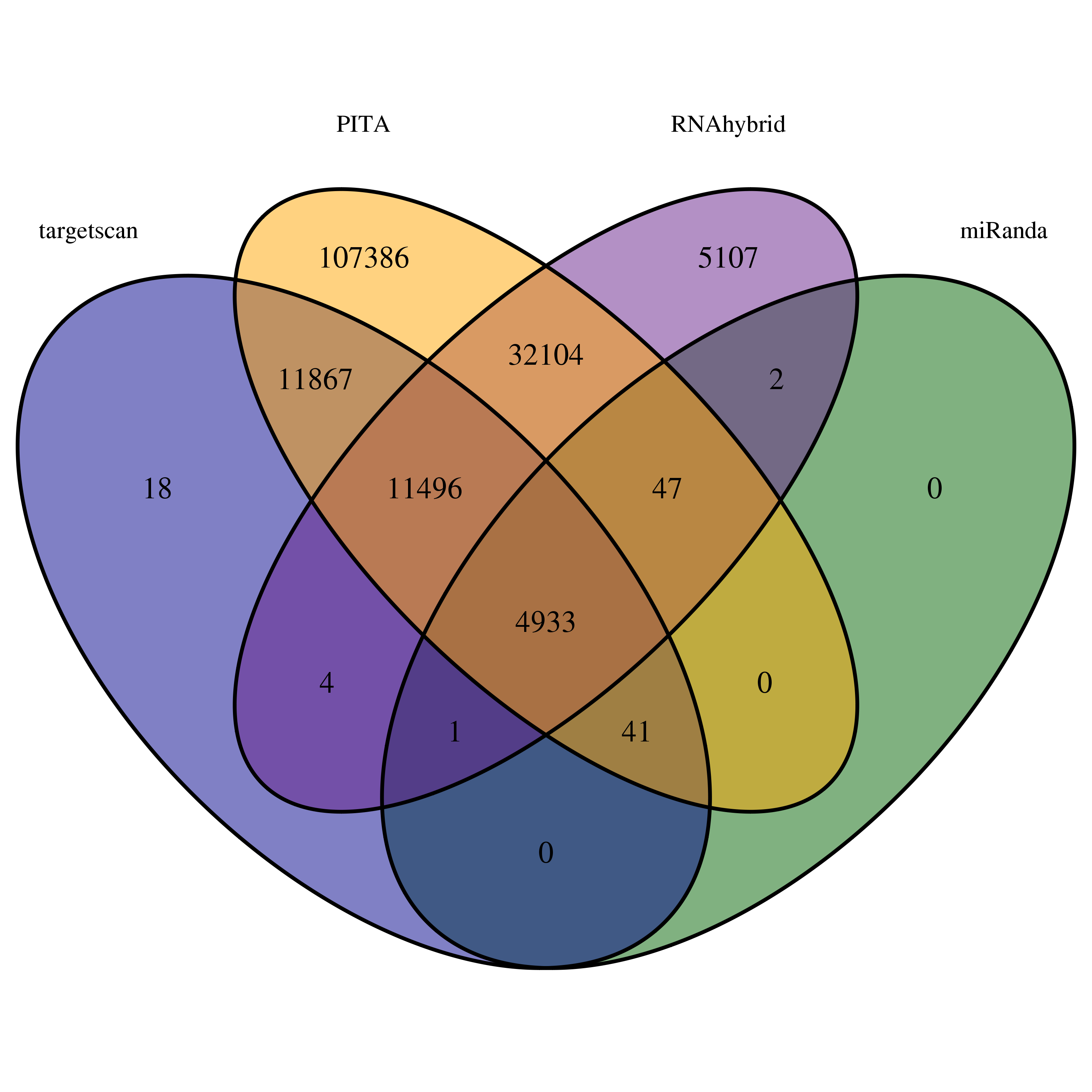

Supplement: Supplemental Information 4 [file peerj-11-15690-s004.zip › Raw data/Figure 3/Figure 3 A.png]

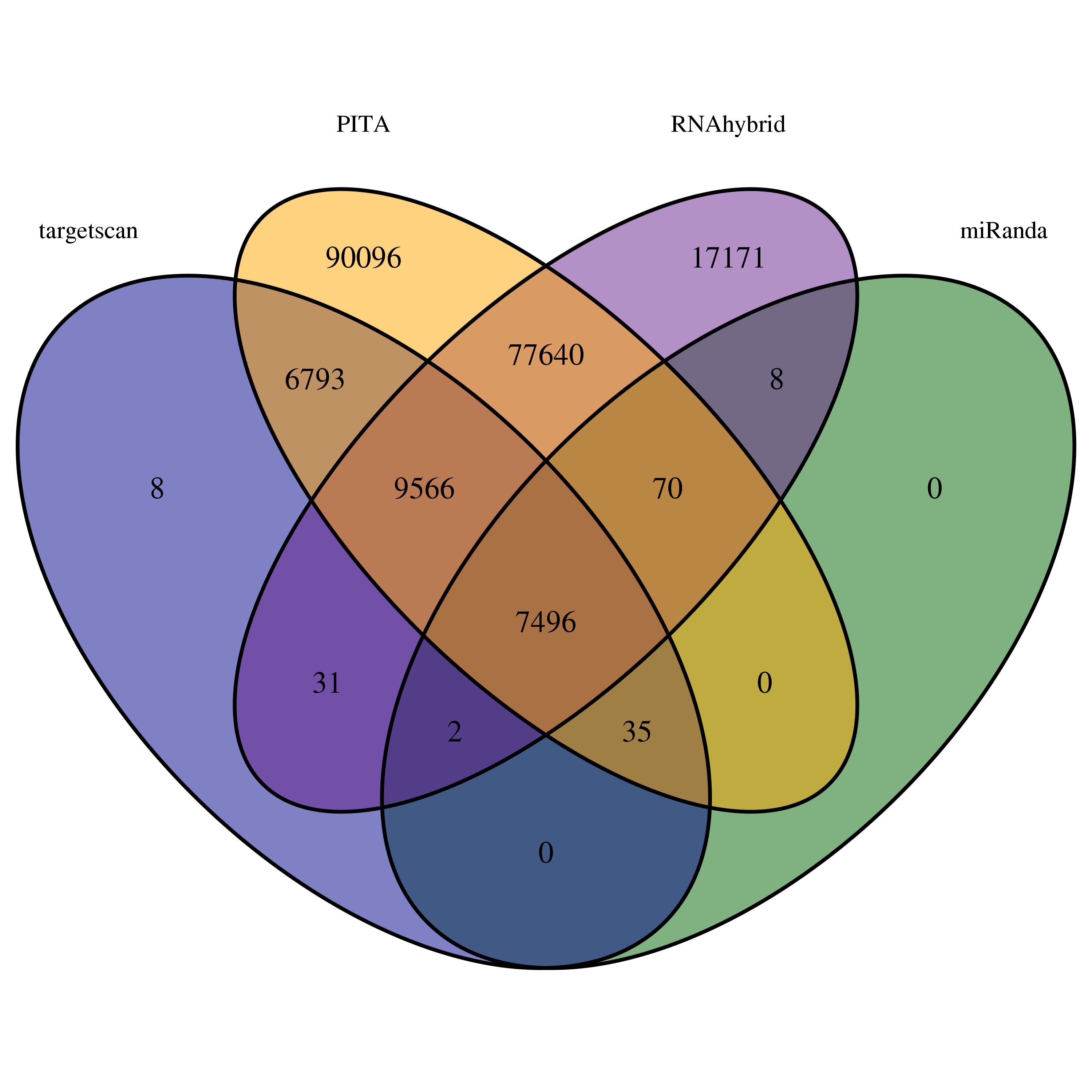

Supplement: Supplemental Information 4 [file peerj-11-15690-s004.zip › Raw data/Figure 3/Figure 3 B.png]

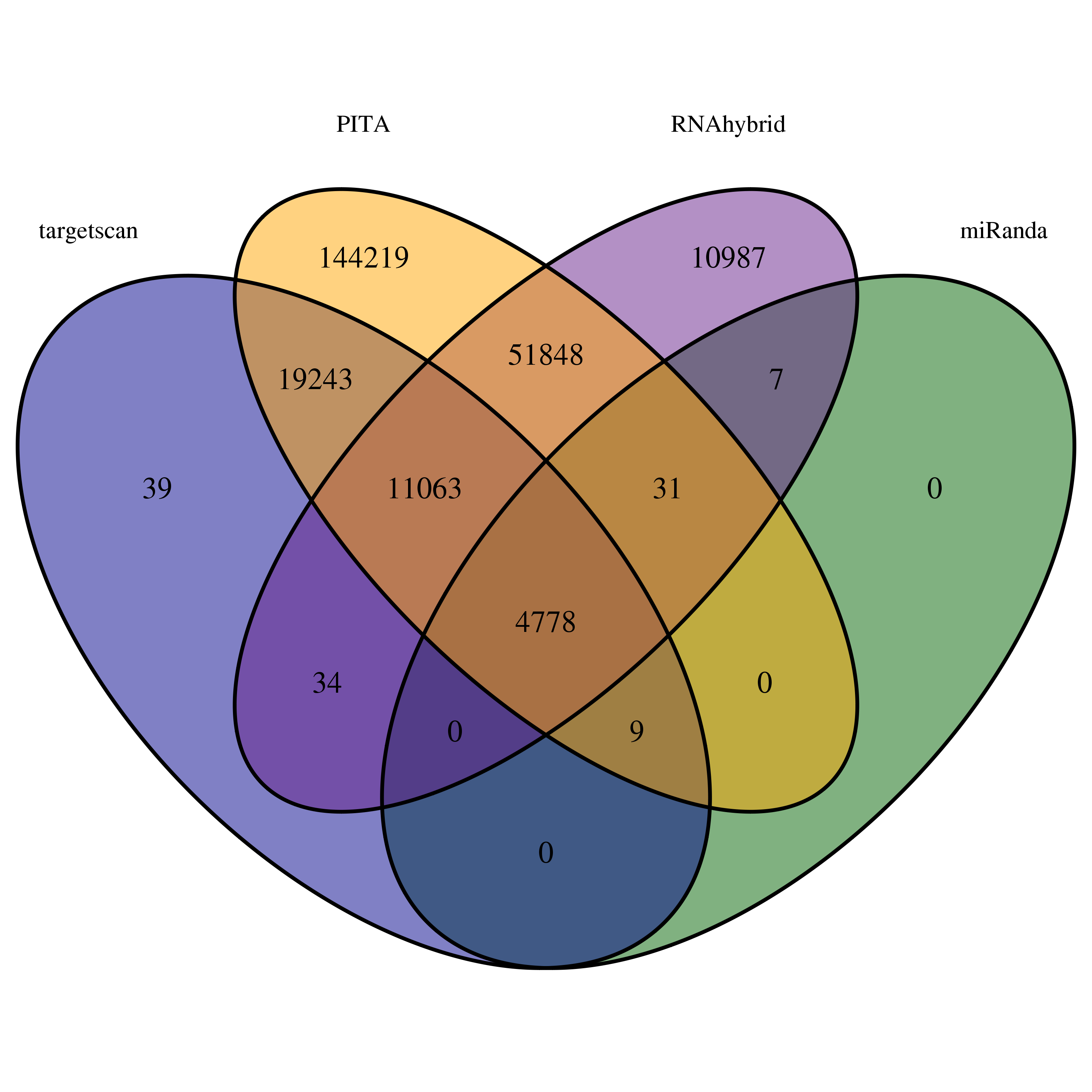

Supplement: Supplemental Information 4 [file peerj-11-15690-s004.zip › Raw data/Figure 3/Figure 3 C.png]

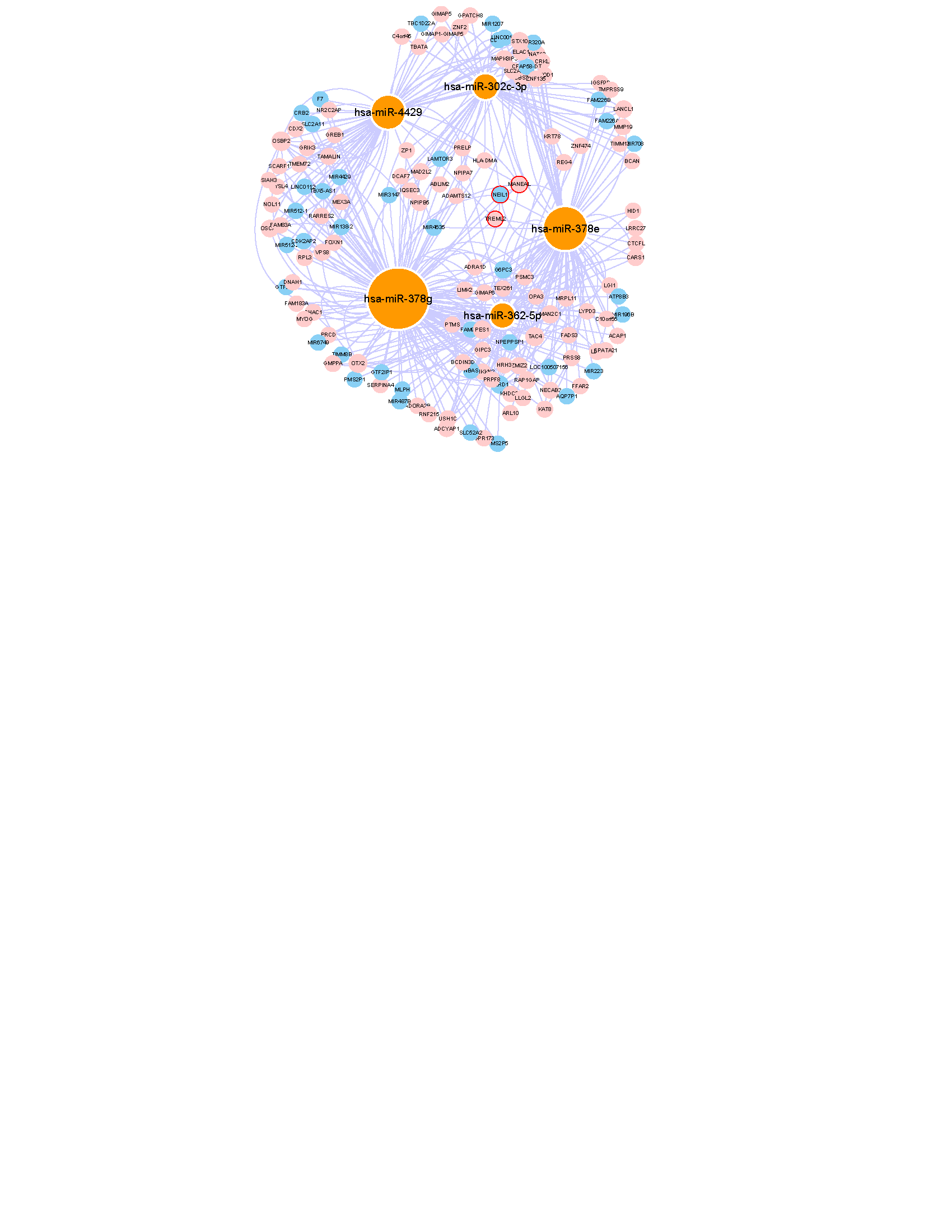

Supplement: Supplemental Information 4 [file peerj-11-15690-s004.zip › Raw data/Figure 4/Figure 4 A.png]

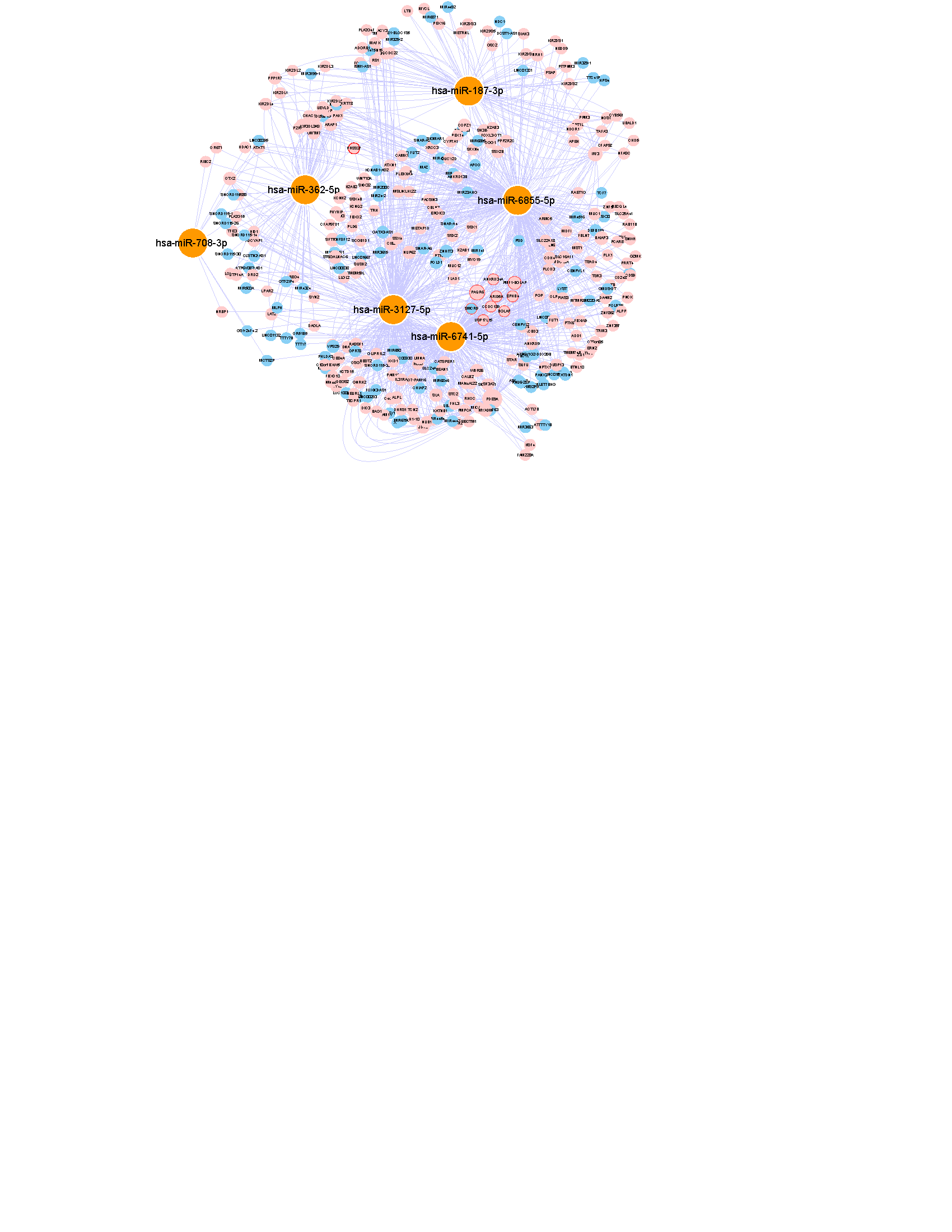

Supplement: Supplemental Information 4 [file peerj-11-15690-s004.zip › Raw data/Figure 4/Figure 4 B.png]

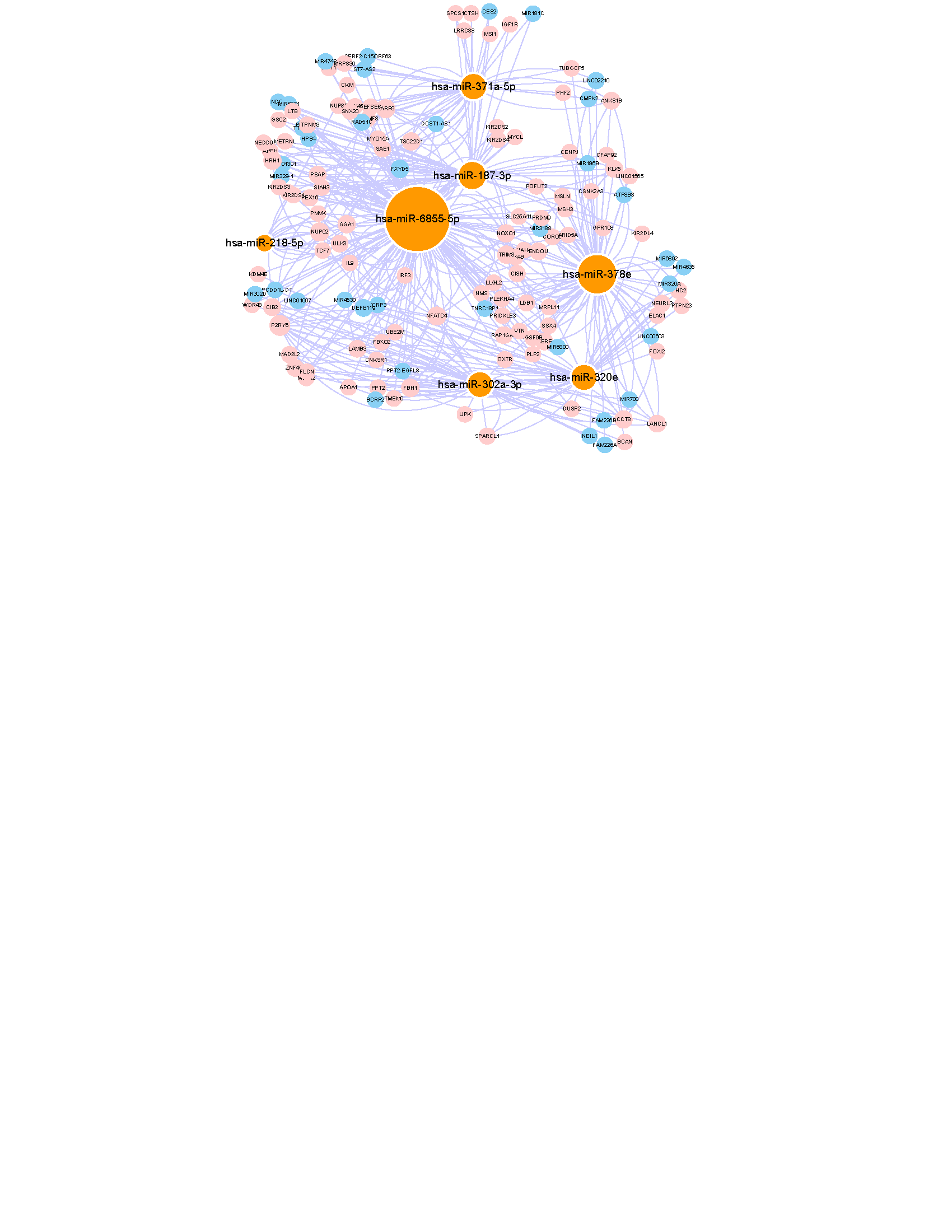

Supplement: Supplemental Information 4 [file peerj-11-15690-s004.zip › Raw data/Figure 4/Figure 4 C.png]

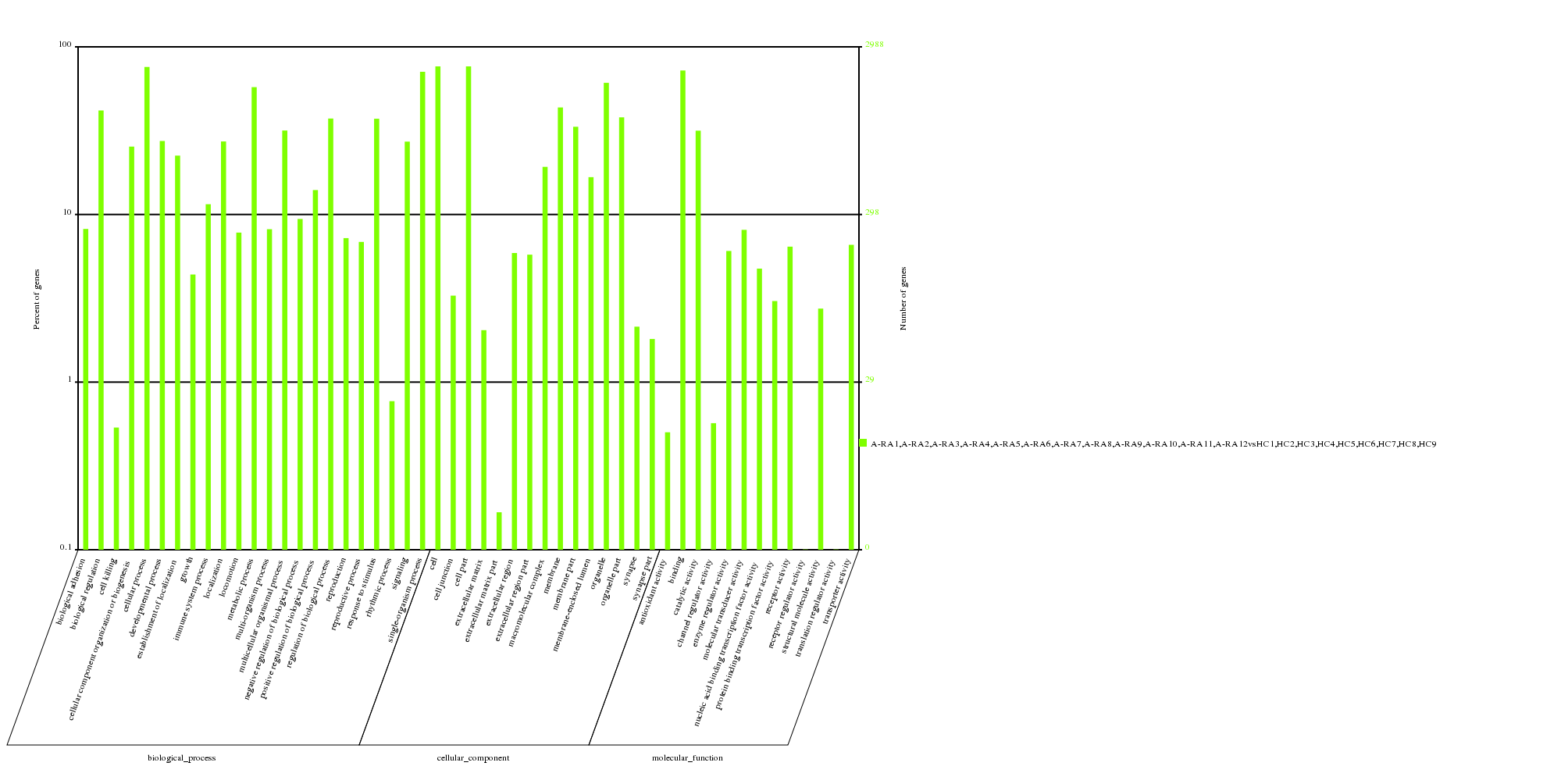

Supplement: Supplemental Information 4 [file peerj-11-15690-s004.zip › Raw data/Figure 5/Figure 5 A.png]

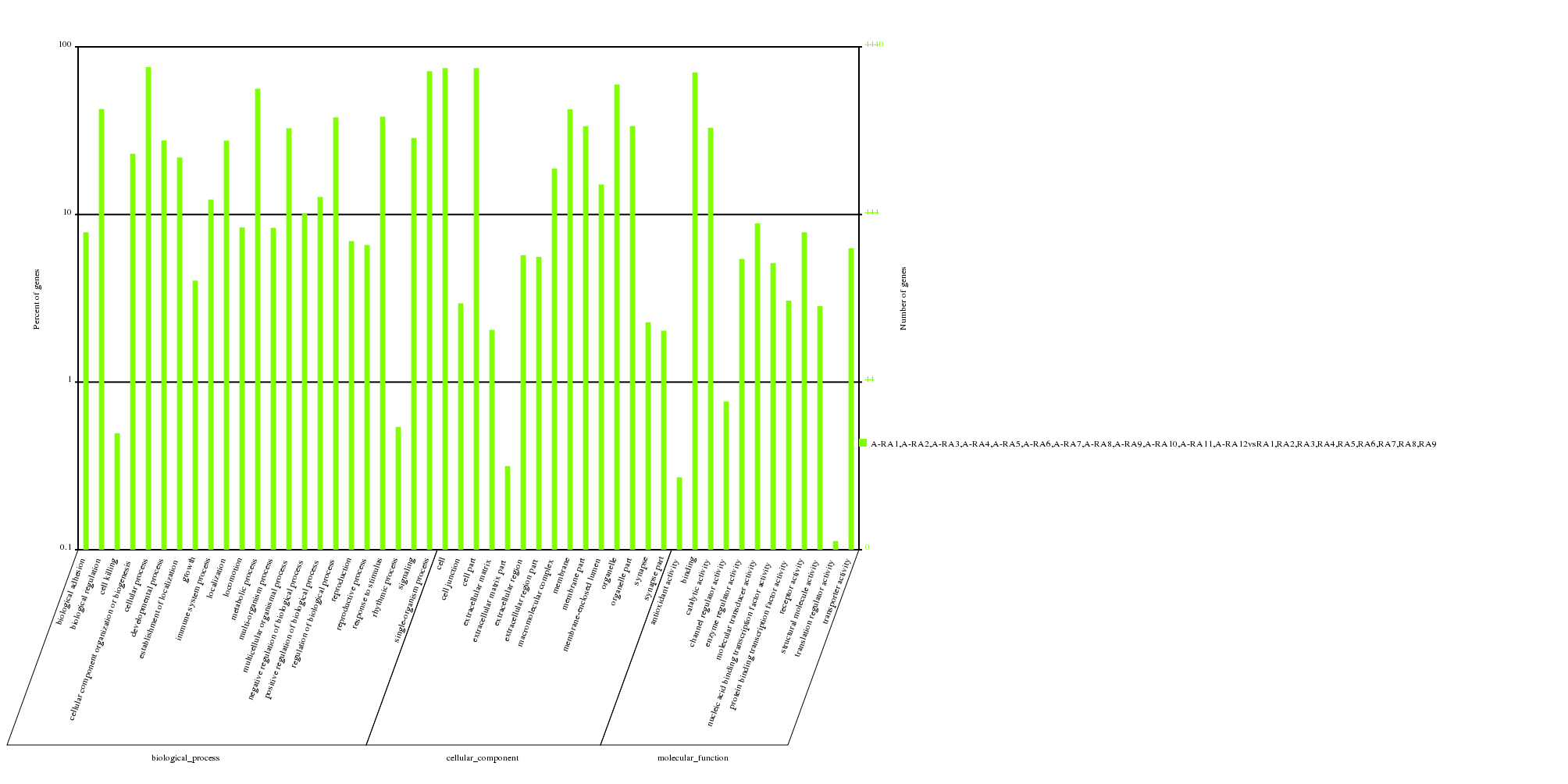

Supplement: Supplemental Information 4 [file peerj-11-15690-s004.zip › Raw data/Figure 5/Figure 5 B.png]

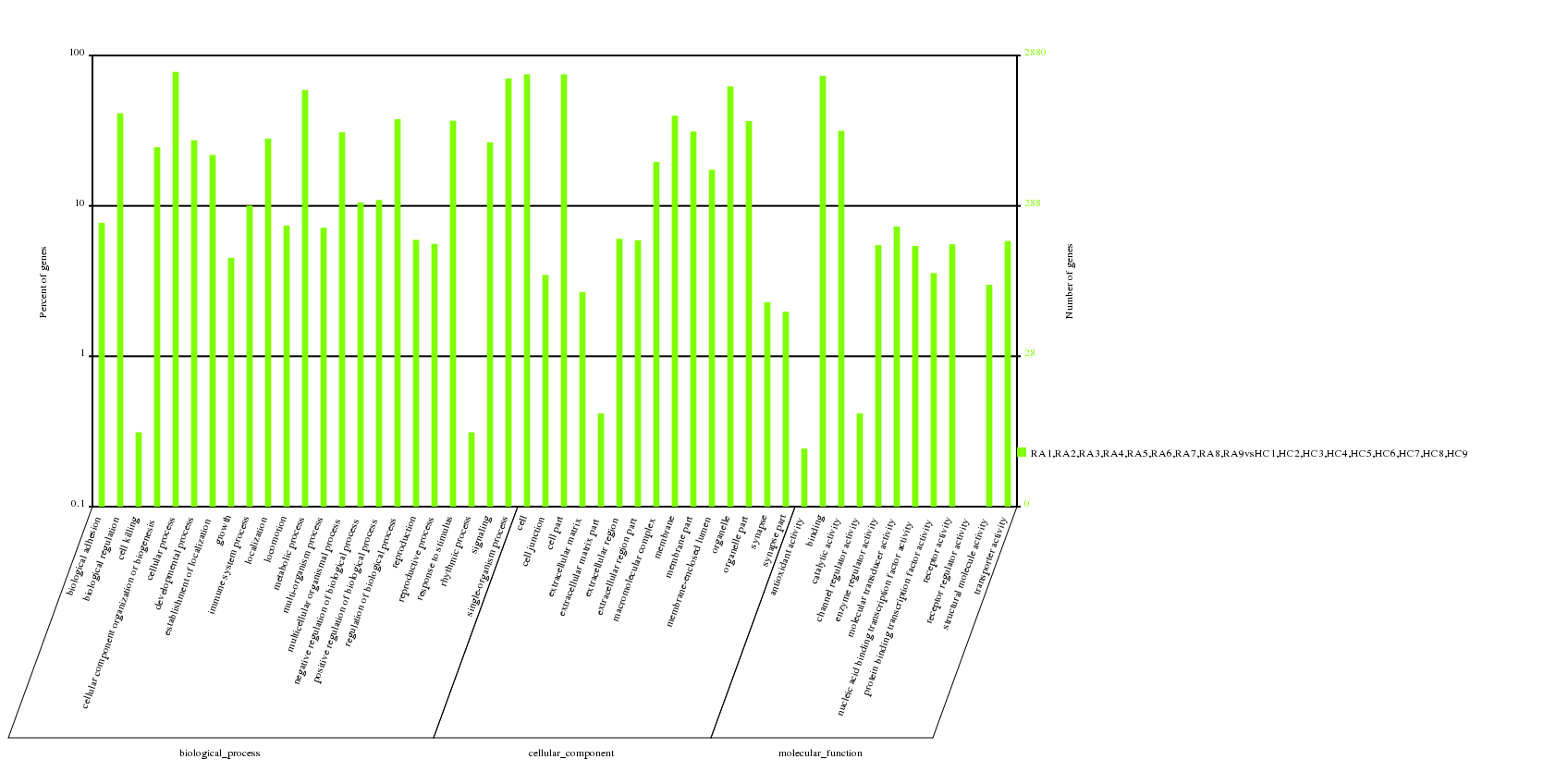

Supplement: Supplemental Information 4 [file peerj-11-15690-s004.zip › Raw data/Figure 5/Figure 5 C.png]

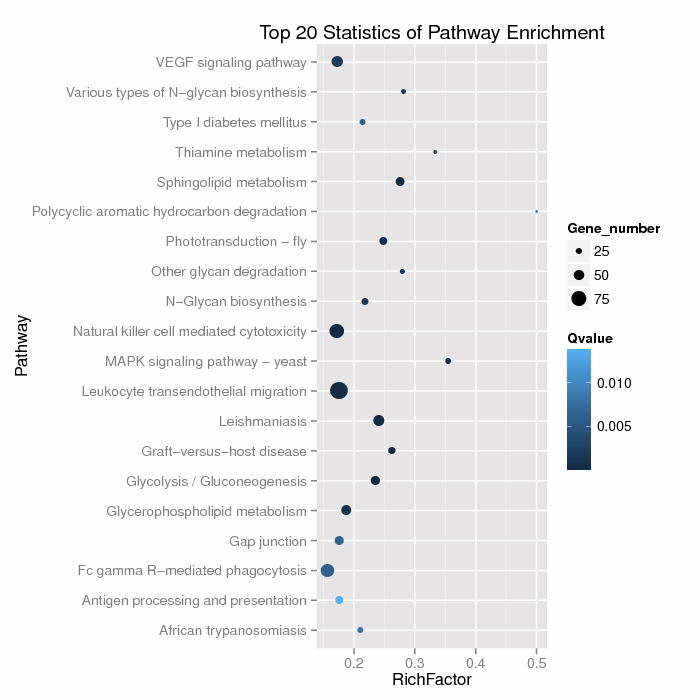

Supplement: Supplemental Information 4 [file peerj-11-15690-s004.zip › Raw data/Figure 6/Figure 6 A.png]

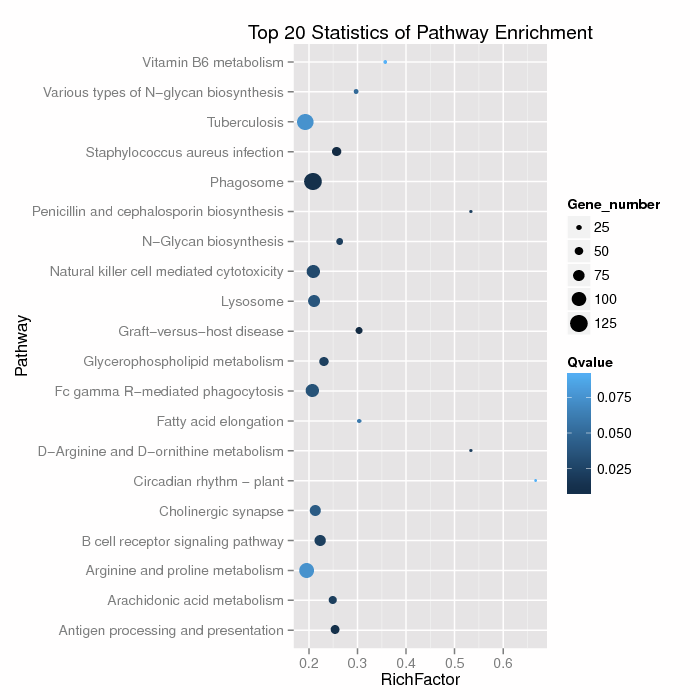

Supplement: Supplemental Information 4 [file peerj-11-15690-s004.zip › Raw data/Figure 6/Figure 6 B.png]

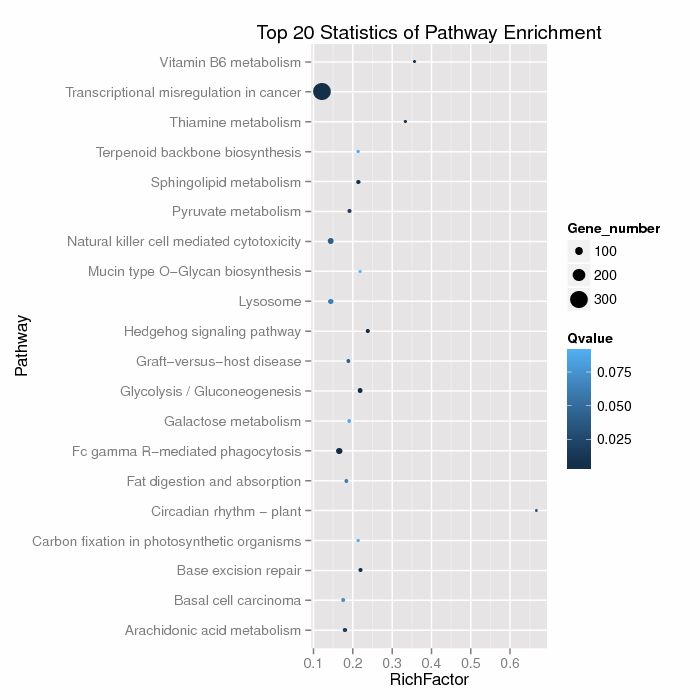

Supplement: Supplemental Information 4 [file peerj-11-15690-s004.zip › Raw data/Figure 6/Figure 6 C.png]
